# Comparing the effectiveness of virtual and semi-attendance Stress Inoculation Training [SIT] techniques in improving the symptoms of anxiety, depression, and stress of pregnant women with psychological distress: a multicenter randomized controlled trial

**DOI:** 10.1186/s12884-023-05650-1

**Published:** 2023-05-12

**Authors:** Atefeh Fatemi, Fatemeh Nasiri-Amiri, Mahbobeh Faramarzi, Mohammad Chehrazi, Hajar Adib Rad, Zeinab Pahlavan

**Affiliations:** 1 Student Research Committee, Babol University of Medical Sciences, Babol, Iran; 2 Social Determinants of Health Research Center, Health Research Institute, Babol University of Medical Sciences, Medical College, Babol, 4717647745 Iran; 3 Social Determinants of Health Research Center, Health Research Institute, Babol University of Medical Sciences, Babol, Iran; 4 Social Determinants of Health Research Center, Department of Biostatistics & Epidemiology, School of Public Health, Health Research Institute, Babol University of Medical Sciences, Babol, Iran; 5 Social Determinants of Health Research Center, Health Research Institute, Babol University of Medical Sciences, Babol, Iran; 6 Department of Obstetrics and Gynecology, Research Development Unit, Ayatollah Rouhani Hospital, School of Medicine, Babol University of Medical Sciences, Babol, Iran

**Keywords:** Stress, Anxiety, Depression, Psychological, Pregnant women, Stress inoculation training

## Abstract

**Background:**

Some studies indicate that more than 10% of pregnant women are affected by psychological problems. The current COVID‐19 pandemic has increased mental health problems in more than half of pregnant women. The present study compared the effectiveness of virtual (VSIT) and semi-attendance Stress Inoculation Training (SIT) techniques on the improvement of the symptoms of anxiety, depression, and stress of pregnant women with psychological distress.

**Methods:**

This study was conducted on 96 pregnant women with psychological distress in a 2-arm parallel-group, randomized control trial between November 2020 and January 2022. The semi-attendance SIT received treatment for six sessions, sessions 1, 3 and 5 as individual face-to-face and sessions 2, 4 and 6 as virtual once a week for 60 min continuously [*n* = 48], and the virtual SIT received six sessions simultaneously once a week for 60 min (*n* = 48) in pregnant women of 14–32 weeks’ gestation referred to two selected hospitals. The primary outcome of this study was BSI-18 [Brief Symptom Inventory] and NuPDQ-17 [Prenatal Distress Questionnaire]. The secondary outcomes were the PSS-14 [Cohen’s General Perceived Stress Scale]. Both groups completed questionnaires measuring anxiety, depression, pregnancy-specific stress, and generally perceived stress questionnaires before and after the treatment.

**Results:**

The post-intervention results showed that the stress inoculation training technique in both VSIT and SIT interventions effectively reduced anxiety, depression, psychological distress, pregnancy-specific stress and general perceived stress [*P* < 0.01]. Also, the SIT interventions on decreasing anxiety [*P* < 0.001, η2 = 0.40], depression [*P* < 0.001, η2 = 0.52] and psychological distress [*P* < 0.001, η2 = 0.41] were more considerable than that of VSIT. However, There was no significant difference between SIT and VSIT intervention in terms of their effects on pregnancy-specific stress [*P* < 0.38, η2 = 0.01] and general stress [*P* < 0.42, η2 = 0.008].

**Conclusion:**

The semi-attendance SIT group has been a more effective and practical model than the VSIT group, for reducing psychological distress. Therefore, semi-attendance SIT is recommended for pregnant women.

## Introduction

Pregnancy, as a critical period of a woman’s life, can be affected by various psychological factors [1]. These physiological factors can lead to various psychological changes in some pregnant women [2]. Approximately 10% of pregnant women experience a mental disorder during pregnancy [3]. The current COVID‐19 pandemic has increased mental health problems in more than half of pregnant women [4, 5]. Mental distress refers to an individual’s experiences of symptoms of anxiety, depression or stress alone or as comorbidity, which is prevalent during the perinatal period, particularly pregnancy [6, 7]. Psychological distress is more commonly reported among women at risk of pregnancy complications [8]. Moreover, it is associated with adverse consequences such as miscarriage [9], preterm delivery [10], hypertension [11], increased birth-related complications, weight loss at birth, long-term effects on the child’s health [12], intrauterine growth restriction [13], and long-term effects on the infant’s cognitive development [14].

Given the fundamental role of maternal health during pregnancy on the long-term health of the developing fetus, it is essential to identify prenatal interventions to reduce maternal distress [15]. Pharmacotherapy and psychotherapy can be used to treat mental disorders during pregnancy. However, due to pregnant women’s distinct conditions and the adverse effects of medications on the fetus and mother’s health, psychological interventions gain priority [16]. One of the most efficient interventions for mental distress is the SIT model, first proposed by Meichenbaum [17]. This method is a precise and multidimensional therapeutic intervention whose goal is not to completely eliminate stress, but rather to encourage clients to consider stressful situations not as a threat to themselves, but as solvable issues. The purpose of this treatment is to help patients to identify, evaluate and correct dysfunctional beliefs and change maladaptive behaviors. The principle of this program is to force people to change their beliefs about stressful behaviors and self-educate about ways to cope with stress. SIT allows repeated practice of coping skills and emotion regulation during a safe and controlled gradually increasing exposure to stressors that cause may at one point cause fear or anxiety. SIT helps distressed individuals become aware of how they can engage in behaviors that maintain and exacerbate their distress. SIT is a flexible, individually tailored, multifaceted form of cognitive-behavioral therapy [16].

A study examining the effect of SIT on pregnant women’s anxiety and sleep disorders concluded that this approach significantly reduced sleep disorders and pregnancy anxiety [18]. In addition, another study that examined this model on pregnant women’s general perceived stress indicated a significant reduction in pregnant women’s stress level [17]. Another study showed that stress inoculation therapy reduced cancer patients’ stress, anxiety, and depression [19]. The results of another study likewise showed that SIT model reduced anxiety in highly stressed students [20]. Similarly, one study found that SIT model reduced perceived stress in women with low birth weight infants [21]. However to our knowledge only one study have reported conflicting results regarding the effectiveness of this method [22]. Notably, this therapeutic method has reduced anxiety, depression, and stress in most cases; however, the number of contrary studies is so small that opposing ideas are trivial in the literature in this regard.

Consequently, further research is needed in this area. Moreover, in addition to face-to-face therapies, today, due to the COVID-19 pandemic and mothers’ reluctance to receive face-to-face therapy regarding the risk of infection, emphasis has been placed on virtual education and therapy. Therefore, the present study aimed to compare the effectiveness of virtual and semi-attendance SIT techniques in improving the symptoms of anxiety, depression and stress in pregnant women with psychological distress.

## Materials and methods

### Design

The present study is a multicenter randomized clinical trial with two parallel intervention groups. It was registered in the Iranian Clinical Trial registry under the number IRCT.20200122046228N1.

### Participant recruitment

The study population consisted of all pregnant women of 14–32 weeks’ gestation referred to selected hospitals. It was performed in two educational and therapeutic centers affiliated with the Babol University of Medical Sciences in Iran between November 2020 and January 2022.

The sample consisted of women meeting the eligibility criteria to enter the study during the trial. A total of 96 individuals who obtained the necessary scores based on the relevant questionnaires were enrolled through the convenience sampling method. Afterward, an independent midwife outside the research team randomly assigned samples into two intervention groups [SIT and VSIT], using the blocking method and computer-generated randomization [23, 24]. Randomization was based on a 1: 1 ratio of four blocks [balanced block randomization] using a computer random number generator [www.random.org]. After the creation of two groups of 4 blocks with 96 participants, 24 random blocks were created using the company computer. The allocation order was kept confidential and was not available to any participant or researcher. A midwife in the selected hospital who was unaware of the treatment allocation and was not involved in the recruitment of the women assessed the consequences. Similarly, the statistical analyzer was not informed about the coding of the semi-attendance intervention and virtual intervention groups.

Inclusion criteria included the willingness to participate in the study, 14–32 weeks of gestation, obtaining a score higher than 0.5 based on the BSI-18 questionnaire, not attending similar classes, no severe psychiatric disorders [such as bipolar] based on self-report, the educational level of at least the third year of middle school, age over 16, and access to the Internet and WhatsApp messenger. Exclusion criteria included inactive and irregular attendance, unwillingness to continue attending meetings, a pregnancy accompanied by high-risk situations [such as bleeding, miscarriage, preterm delivery] and migration.

### Sample size calculation

The sample size was calculated at 48 participants for each group using G-Power and Mohammadi et al.’s article [21].

First type errors (α):0.05 s type error (β): 0.1

$$\begin{array}{cc}\begin{array}{ccc}\upmu 2:42.02& \mathrm{S}1:9.01& \mathrm{S}2:8.64\end{array}&\upmu 1:34.11\end{array}$$

$$\mathrm{n }= \frac{{\mathrm{S}}_{1}^{2}+{\mathrm{S}}_{2}^{2}}{{\left({\upmu }_{2}-{\upmu }_{1}\right)}^{2}}\mathrm{f}\left(\mathrm{\alpha },\upbeta \right)$$

To implement the intervention, the researcher first provided the necessary explanations about the study objectives to the subjects and obtained their informed written consent if they agreed to participate in the study. Participants were also reassured about the right to withdraw from the study in case of reluctance to participate.

### Measurements

The demographic and fertility information questionnaire included the mother’s age, job, and education level, the father’s education level and job, the family’s economic status, place of residence, gestational age, number of pregnancies, number of living children and history of infertility.

The primary outcome of this study was anxiety, depression and psychological distress by Brief Symptom Inventory [BSI-18] and Revised Prenatal Distress [NuPDQ] were used.

BSI-18 [Brief Symptom Inventory] assesses three factors: depression, anxiety and somatization. Scoring is based on a five-point Likert scale. [0 = not at all, 1 = a little bit, 2 = moderate, 3 = quite a bit, 4 = extremely]. Pregnant women with a score higher than 0.5 were diagnosed with psychological distress and entered the study. The validated Persian version was used in the study that Reliability coefficients and retest coefficients were 0.90 and 0.81 respectively [25].

NuPDQ-17 [Prenatal Distress Questionnaire] includes 17 items and three subscales. The subscales include 1- concerns about giving birth and the infant, 2- concerns about body image/weight and 3- concerns about relationships and emotions. Participants’ responses are scored on a 3-point Likert scale [0 = not at all, 1 = somewhat, 2 = very much] with a cut-off of 16. Reliability coefficients [Cronbach’s alpha] for 3 Persian NuPDQ-17 subscales were 0.73 to 0.93. The total Cronbach’s alpha of this instrument was 0.78 [26].

The secondary outcome of this study was the general stress of pregnant women measured by the Perceived Stress Scale [PSS-14]. PSS-14 [Cohen’s General Perceived Stress Scale]; This tool consists of 14 items which are all measured scored on a 5-point Likert scale from [Never = 0, almost never = 1, sometimes = 2, often = 3 and very often = 4], [27]. PSS-14 has a possible range of scores from 0 to 56. The scale has been reported to have good psychometric properties in the Iranian population. A higher score indicates greater levels of perceived stress. Its Cronbach coefficient was 0.81 [28].

Before initiating the therapy, all pregnant women completed a demographic information questionnaire, BSI-18 questionnaire, NuPDQ-17 Prenatal Distress Questionnaire and General Perceived Stress Questionnaire [PSS-14] with the assistance of an expert outside the research team.

### Interventions

The sessions are six consecutive sessions, one day a week. The content of the sessions is the same for both groups. The structure of SIT therapy sessions was described in Table 1.

**Table 1:** Outline of the training sessions

Training sessions	Description of training sessions
First session	Introduction and explanation of objectives, learning about pregnancy anxiety, conceptualizing the SIT model, learning about thoughts or ideas formed in our minds, recognizing automatic thoughts and starting to change them, identifying positive and negative thoughts, using the first four steps of recording thought changes, and assigning homework.
Second session (virtual)	Recognizing cognitive distortions, modifying automatic thought, evidence review, creating logical alternatives to automatic thoughts, and assigning homework.
Third session	Learning about various methods of coping with stress, emotion-oriented coping, problem-oriented coping, and assigning homework.
Fourth session (virtual)	Physical relaxation training, assigning homework, and practicing physical relaxation at home.
Fifth session	Self-expression skills training, assigning homework, and practicing physical relaxation at home.
Sixth session (Virtual)	Exercising the previously learned skills in the training session and gradually in real-life events, especially in stressful situations.

#### SIT group

The session outline for intervention group 1 was as follows: SIT technique was conducted by a female expert psychotherapist (author MF) who had a license in psychology. The psychologist and Perinatologist (author ZP) organized weekly 60-min face-to-face group sessions for a period of 6 weeks. A female assistant (author AF), who was trained in SIT technique before the trial, helped the therapist in the sessions. sessions 1, 3 and 5 as individual face-to-face and sessions 2, 4 and 6 as virtual SIT held on WhatsApp messenger. Each session lasted for 60 min.

#### VSIT group

Intervention VSIT group subjects received six sessions of virtual SIT treatment simultaneously. Virtual group meetings were held on WhatsApp messenger through which podcasts [consultant’s voice messages], videos, similar examples in PDF format and relaxation audio files were sent. To receive a consultation, the first researcher contacted participants individually every week for at least 30 min.

**Fig. 1:**
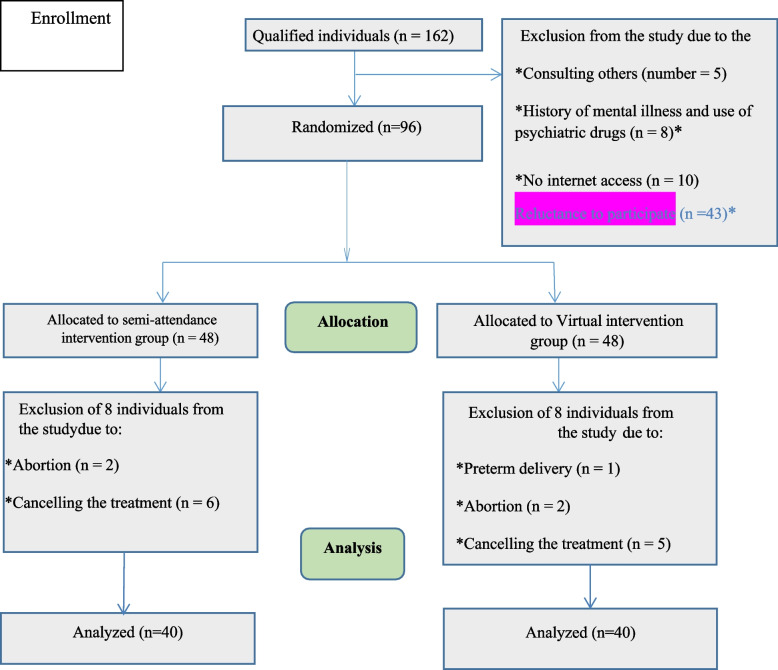
Flow-Chart of the Participants

During the week, a supportive voice message was prepared and sent to subjects to maintain contact with them. Furthermore, the first researcher contacted both groups weekly to prevent sample loss. All study subjects completed the BSI-18, NuPDQ-17 and PSS-14 questionnaires after six weeks of intervention. The primary outcomes of this intervention were anxiety, depression and pregnancy-specific stress and the secondary outcome was perceived stress. In the semi-attendance intervention group, two patients were excluded due to abortion and severe bleeding and six patients withdrew from the study due to reluctance to continue treatment. One and two patients were excluded from the virtual intervention group due to preterm delivery and abortion respectively. Moreover, five participants withdrew from the study due to unwillingness to continue treatment. Finally, 80 patients were analyzed (Fig. 1).

### Data management and analysis

The mean and the relevant standard deviation for presenting patients’ characteristics and the study outcomes were used as summary measures in tables and results. An intention-to-treat analysis was used to manage the missing outcomes. Multiple Imputation technique was applied for addressing the missing observations. MI Impute Chained (MICE) was chosen as an iterative process. Sixteen imputations of variables with missing values on the observed dataset were added. A multiple regression approach for analysis of covariance (ANCOVA) was used to estimate the differences of scores between pre-intervention and post-intervention phases in the two groups. Pre-test scores were considered as a covariate variable and trial intervention as the fixed factor. Also, partial eta squared (η^2^) was employed to examine the effect sizes. We defined the effect sizes as small (η^2^ = 0.01), medium (η^2^ = 0.06) and large (η^2^ = 0.14) effects according to Cohen (1988), [29]. All analyses were conducted using SPSS software (Version 26). The significance level was less than 0.05.

#### Ethical considerations

The trial was approved by the Ethics Committee of the National Institute [IR.MUBABOL. HIR.REC.1399.277] and registered in the IRCT**-** [IRCT20200122046228N1**].** In order to do ethical consideration, this study followed the guidelines set by declaration of Helsinki and received ethical approval for human subject by the Ethics Committee of Babol University of Medical Sciences approved the study. The report was provided based on the Consolidated Standards of Reporting Trials (CONSORT) guidelines. Informed consent was obtained from all participants.

## Results

The demographic, fertility and social characteristics statistics are presented in Table 2. There were no significant differences in participants’ age, education, employment status, residency status, and husband's education and job.

**Table 2:** Demographic, social, and fertility characteristics of participants in the two groups(*N* = 80)

Varables	Virtual SIT Number (%)	semi- attendance SIT Number (%)	*P* value
Age (years), Mean (SD^*^)	28.20 (5.50)	29.97(5.77)	0.964
Education, n (%)			
Below diploma	1(2.5)	3(7.5)	0.481
Diploma	17 (42.5)	14(35)	
University	22 (55)	23 (57.5)	
Husband's Education, n (%)			
Below diploma	8(20)	8 (20)	0.525
Diploma	15 (37.5)	16(40)	
University	17 (42.5)	16 (40)	
Place of residence, n (%)			
City	23 (57.5)	26 (65)	0.491
Village	17 (42.5)	14 (35)	
Job, n (%)			
Housewife	31 (77.5)	35 (87.5)	0.434
Employed	9 (22.5)	5 (12.5)	
Husband's Job, n (%)			
Freelancer	34 (85)	31(77.5)	0.491
Governmental	6 (15)	9 (22.5)	
Gestational age (week), Mean (SD^*^)	19.7(5.1)	22.6(5.8)	0.162
Gravida, Mean (SD^*^)	2.1(1.0)	2.2(1.0)	0.830
Number of children, Mean (SD^*^)	0.6(0.6)	0.7(0.6)	0.707
Number of abortions, Mean (SD^*^)	0.5(0.7)	0.6(0.8)	0.769
History of infertility, n (%)			
Yes	3(7.5)	1(2.5)	0.305
No	37(92.5)	39(97.5)	
unwanted pregnancy, n (%)			
Yes	8(20)	6(15)	0.556
No	32(80)	34(85)	

^*^SD Standard deviation

^*^T-test was used for quantitative variables (mean and standard deviation) and chi-square test was used for qualitative variables (number and percentage)

According to Table 2, the two study groups were homogeneous regarding the gestational age, number of gravida, children, and abortions. The majority of participants in both groups had intended pregnancies and not history of infertility.

The mean and standard deviation of anxiety, depression, psychological distress, pregnancy-specific stress and general perceived stress of the studied population in the two groups before the intervention did not have significant statistical differences. According to Table 3 and considering the analysis of covariance [ANCOVA] and eliminating the possible confounding effect of scores before the intervention, it was found that the effect of SIT intervention and VSIT intervention on anxiety, depression, pregnancy-specific stress and general perceived stress was significant. According to the mean scores after the intervention, it can be concluded that the mean score of pregnant women’s anxiety [*P* < 0.001, η^2^ = 0.40], depression [*P* < 0.001, η^2^ = 0.52] and mental distress [*P* < 0.001, η^2^ = 0.41], were lower following SIT intervention than VSIT intervention. However, there was no significant difference between SIT and VSIT intervention in terms of their effects of pregnancy-specific stress [*P* < 0.38, η^2^ = 0.01] and general stress [*P* < 0.42, η^2^ = 0.008]. Levene's test was not significant in all variables *P* > 0.05. So the assumption of equality of variances has been established.

**Table 3:** Comparison of mean anxiety, depression, psychological distress, specific stress, general Perceived Stress of women before and after the intervention (*N* = 80)

Group* Outcome*	Virtual SIT		semi-attendance SIT		*P*-value	effect size( η^2^)	Adjusted R Squared
	Before Mean ± SD^*^	After Mean ± SD	Before Mean ± SD	After Mean ± SD			
Anxiety	6.0 ± 4.1	6.2 ± 4.7	4.5 ± 3.7	3.2 ± 3.3	< 0.001	0.40	0.46
Depression	5 ± 4.4	5.3 ± 4.6	2.9 ± 2.8	2.0 ± 2.7	< 0.001	0.52	0.59
Psychological distress	0.9 ± 0.4	0.9 ± 0.6	0.7 ± 0.3	0.5 ± 0.4	< 0.001	0.41	0.47
Specific pregnancy stress	12.4 ± 5.8	12.1 ± 7.0	11.0 ± 5.7	9.9 ± 6.5	0.38	0.01	0.62
General Perceived Stress	24.8 ± 8.9	22.7 ± 8.1	19.4 ± 9.3	19.5 ± 7.5	0.42	0.008	0.73

*SD Standard deviation

*η*^*2*^ partial eta squared

The number of eligible pregnant women who did not participate in the study for any reason was identical in both groups. Statistical analysis was performed by intention-to-treat analysis [ITT] and there was no statistical difference between the two groups before and after the intention-to-treat analysis.

## Discussion

The present study was conducted to compare the effectiveness of virtual and semi-attendance Stress Inoculation Training on improving the symptoms of anxiety, depression and stress in pregnant women with mental distress. So far, this method has not been used to compare groups virtually or as semi-attendance. The results showed that anxiety, depression, pregnancy-specific stress and general perceived stress in pregnant women of both groups decreased after the intervention. This treatment reduced anxiety, depression, psychological distress in SIT intervention compared to VSIT intervention. There was no significant difference between SIT and VSIT intervention in terms of their effects on pregnancy-specific stress and general stress.

Anxiety is one of the most common accompanying symptoms in pregnant mothers, which causes various problems for both mother and fetus. This technique can reduce pregnant women’s misconceptions that lead to anxiety. The results of this study were in line with the study by Jokar et al. They showed that Stress Inoculation Training reduced anxiety in pregnant women [18]. Studies have shown that stress management reduces pregnancy anxiety after intervention [30, 31]. These findings are also consistent with Bersamin’s findings on the effect of SIT on student anxiety [20] and Abdi’s findings on anxiety before the competition [9]. The results of Jolstedt et al.'s study showed statistically significant changes after virtual therapy for anxiety and depression for children with anxiety disorders [32]. Also, the results of Lunkenheimer et al.'s study include the effectiveness of internet and mobile phone-based CBT on depression and anxiety symptoms [33]. By using SIT techniques, we correct false beliefs that lead to anxiety so maybe this is the reason for reducing women's anxiety. On the other hand, depression and anxiety in pregnant women due to their irrational thoughts and beliefs cause numerous problems and expose them to mental and physical hazards during pregnancy. Coping skills included in SIT allow individuals to deal with stressful situations innovatively and assess their previous beliefs.

Askari et al., in their research, showed that positive psychoeducation training and SIT reduced depression in pregnant women [34]. The results of this study are consistent with the present study. Likewise, the present study results are in line with Kashani’s study, which examined the effect of SIT on stress, anxiety and depression in cancer patients [19]. In Jabbari’s study, the experimental group’s mental status mean scores in the post-test stage were significantly lower than the mean scores in the control group. The results of this study are consistent with the present study [35]. In the study of Shafirizi et al. At end of-treatment, improvements in ICBT were non-inferior to CBT for symptoms of anxiety and depression [36]. The results of Compen et al.'s study in face-to-face and online mindfulness intervention led to a statistically significant and clinically reliable reduction of psychological distress compared to conventional treatment [37]. In one clinical trial, Wagner et al. reported high and equal effectiveness in reducing depression scores in both CBT and ICBT [38]. The reason why SIT technique leads to depression reduction is that in this technique with coping skills program, how to use and receive social support is taught and this process helps to reduce depression in these people. People's positive inner perception of themselves helps control mental pressure and, as a result, reduce depression caused by it.

Stress is one of the most common concerns among pregnant women directly related to fetal developmental processes that cause irreversible effects on the fetus. The present study results are consistent with Khorsandi et al.’s study conducted to investigate the effect of Stress Inoculation Training on pregnant women’s perceived stress. They showed that SIT could significantly reduce stress in pregnant women [17]. Also the results of the present study are in line with the study by Navidian et al. and the study by Hassanzadeh et al. They showed that stress mean scores decreased after the intervention [39, 40]. Lif Boß et al.'s study showed evidence of greater effectiveness of internet-based stress management compared to face-to-face group stress management [41]. The results of this study were contrary to our study. Because in our study, semi-attendance SIT and virtual SIT techniques were equally effective in reducing stress. In this regard, the study by Urech et al. showed no significant difference between the two groups in terms of birth outcome, psychological and biological parameters, both internet intervention and conventional treatment showed equivalent effects and were useful for psychological, social and well-being distress [42]. In the recent study, the specific stress of pregnancy and general stress did not have a statistically significant difference in the two SIT techniques, maybe it is because of the covid-19 pandemic and the stress caused by it that both methods were able to reduce stress equally.

### Limitations of the study

This study had strengths and limitations that should be noted. One of the main strengths is that the present study was the first randomized clinical trial to compare the effectiveness of Virtual with face-to-face Semi-Attendance SIT techniques in Pregnant Women with Psychological distress for improving the symptoms of anxiety, depression and stress. In addition, we used validated scales to assess primary and secondary outcomes. Another strength of the randomized clinical trial design was the blinding of the outcome assessor and statistician to the type of intervention.

This study has some limitations, which demand caution for the generalization of the results. Firstly, to evaluate depression, anxiety and stress in pregnant women, Self-report scales were used. It is suggested that in future studies, a clinical interview be implemented by an expert since it can be a better indicator. Secondly, we did not measure the effect of SIT intervention on pregnancy outcome. It is recommended to measure the effect of this method on improving maternal and newborn outcomes. Also, the lack of a passive control group made the relative effectiveness of the intervention against the passive intervention not investigated.

## Conclusion

The results indicated that anxiety, depression and stress in pregnant women decreased after the intervention by virtual and semi-attendance SIT techniques. The SIT intervention has been a more effective and practical model than the VSIT intervention for reducing psychological distress. Therefore, this model is recommended for pregnant women.

## Data Availability

The data sets used and analyzed during the current study are available from the corresponding author on reasonable request.
